# Shared and distinct changes in the molecular cargo of extracellular vesicles in different neurodegenerative diseases

**DOI:** 10.1007/s00018-024-05522-7

**Published:** 2024-12-03

**Authors:** Anna F. Wiersema, Alyssa Rennenberg, Grace Smith, Suzy Varderidou-Minasian, R. Jeroen Pasterkamp

**Affiliations:** Department of Translational Neuroscience, University Medical Center Brain Center, Utrecht University, Utrecht, The Netherlands

**Keywords:** Neurodegeneration, Extracellular vesicles, Alzheimer’s disease, Parkinson’s disease, Amyotrophic lateral sclerosis

## Abstract

Neurodegenerative disorders such as Alzheimer’s disease (AD), amyotrophic lateral sclerosis (ALS) and Parkinson’s disease (PD) affect millions of people worldwide. Curative treatment for these neurodegenerative disorders is still lacking and therefore a further understanding of their cause and progression is urgently needed. Extracellular vesicles (EVs) are nanosized vesicles loaded with cargo, such as proteins and miRNAs, that are released by cells and play an important role in intercellular communication. Intercellular communication through EVs can contribute to the spread of pathological proteins, such as amyloid-beta and tau, or cause pathogenesis through other mechanisms. In addition, EVs may serve as potential biomarkers for diagnosis and for monitoring disease progression. In this review, we summarize and discuss recent advances in our understanding of the role of EVs in AD, ALS an PD with an emphasis on dysregulated cargo in each disease. We highlight shared dysregulated cargo between these diseases, discuss underlying pathways, and outline future implications for therapeutic strategies.

**Supplementary Information:**

The online version contains supplementary material available at 10.1007/s00018-024-05522-7.

## Introduction

**Fig.1:**
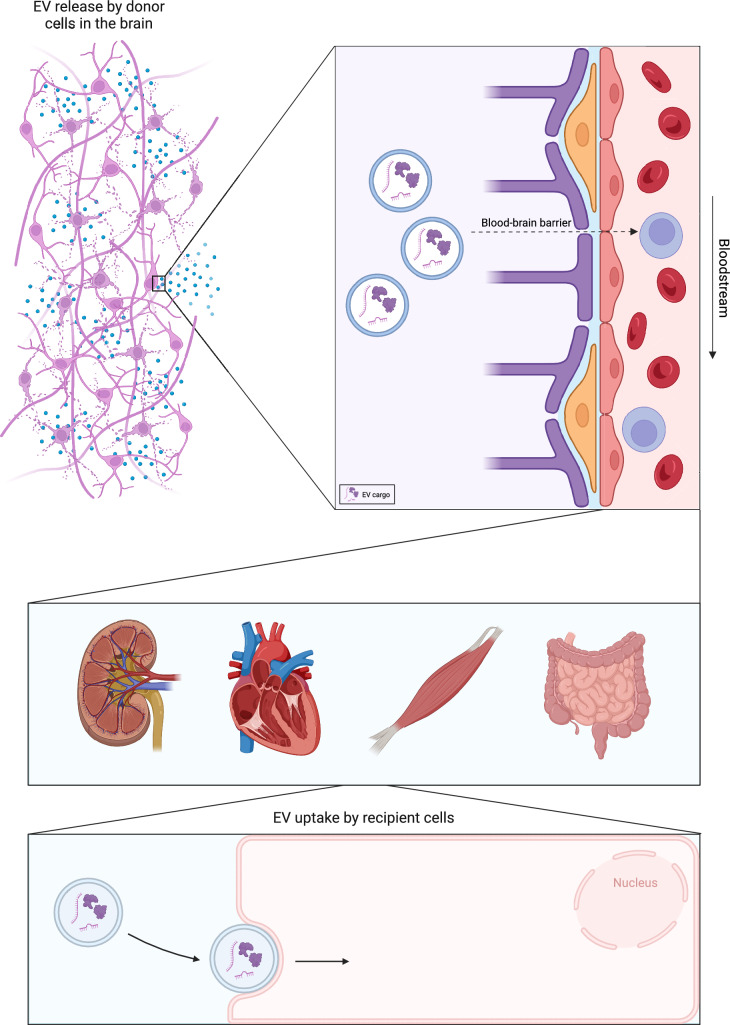
Transport of EVs from the brain to other parts of the body. Schematic overview showing the path of EVs from the brain (neurons and glial cells) through the blood–brain-barrier (BBB) to other parts of the body. EV biogenesis occurs in various neural cell types and is followed by secretion into the extracellular space. EVs can subsequently travel through the BBB and enter the bloodstream. Consequently, EVs reach various organs and tissue types throughout the body and release their cargo in recipient cells. Depending on the cargo, EV molecules can activate pathways such as apoptosis, inflammation and autophagy

Progressive neurodegenerative disorders such as Alzheimer’s disease (AD), amyotrophic lateral sclerosis (ALS), and Parkinson’s disease (PD) affect millions of people worldwide. Although these disorders are highly heterogeneous in their etiology and symptoms, they share progressive neuron loss resulting in cognitive and motor problems [1–3]. As these disorders lack curative treatment, further insight into their underlying disease mechanisms is needed. A key feature of many neurodegenerative disorders is the exchange of pathogenic molecular factors between cells, often as cargo of extracellular vesicles (EVs). EVs are nanosized vesicles consisting of a lipid bilayer secreted by all eukaryotic cells [4]. EVs can be subcategorized into exosomes (50–100 nm), microvesicles (100–1000 nm) and apoptotic bodies (100–5000 nm). Exosomes are released through the endolysosomal pathway, whereas microvesicles and apoptotic bodies are released by direct budding of the plasma membrane [5, 6]. EVs are loaded with cargo such as proteins and miRNAs and play an important role in intercellular communication [7, 8]. In addition, neurons and glial cells release EVs into the extracellular space and these vesicles can pass the blood–brain-barrier (BBB) and enter the bloodstream (Fig. 1). Once in the circulation, brain-derived EVs can be filtered by the kidneys to be excreted in urine. These peripheral trajectories allow the analysis of brain-derived EVs from non-invasive sources such as serum and urine. Specific molecular signatures present in EVs, such as altered microRNA profiles, can serve as biomarkers for neurodegenerative disease states or help identify potential therapeutic targets [9]. Further, in neurodegenerative disorders such as AD, ALS and PD it has been shown that pathological proteins such as tau, transactive response DNA-binding protein 43 (TDP-43) and α-synuclein can be packaged into EVs. EVs may contribute to the spread of these proteins between cells, thereby promoting disease propagation [10–12].

Several isolation techniques have been developed to acquire pure EV populations from various types of tissue [5, 13]. The most widely used technique is ultracentrifugation, which allows the isolation of EVs from large volume samples. Another commonly used method is size exclusion chromatography (SEC), which separates EVs based on their size with minimal EV damage. In addition, an increasing number of commercial EV isolation kits have been developed, which use a variety of approaches such as magnetic beads with affinity for EV-markers such as CD9, CD63 or CD81 and columns containing specialized affinity membranes to precipitate EVs [14]. To isolate pure neuron-derived EV (NDEV) populations, subsequent isolation steps are needed when using blood or serum samples instead of postmortem brain tissue. For example, L1 adhesion molecule (L1CAM) is commonly used as a neuron-specific marker [15]. However, a recent study has questioned the effectivity of L1CAM-based immunoaffinity and emphasizes the need for more specific markers to acquire high yields of pure NDEVs. Therefore, it is important to note that EV isolation techniques are still lacking a gold-standard method for acquiring pure NDEV populations derived from blood and serum samples [16]. EV purification methods have allowed detailed characterization of EV cargo. Multi-omics studies report different EV proteomes, transcriptomes and lipidomes providing insight into disease-associated pathways and potential biomarkers [17]. Especially, exosome cargo is of great interest when studying neurodegeneration because of their release by the endolysosomal pathway. In this review, we highlight changes in the molecular cargo of EVs (RNA and protein) in the context of neurodegenerative disorders (AD, ALS and PD) and discuss the potential implications of these changes based on known or predicted pathways linked to the specific cargo. It is important to note that implications based on changes in EV content are only valid if they reflect altered levels of the detected RNAs or proteins in the donor cell or lead to altered levels and downstream pathway activity in recipient cells. For many observations further experimental work is needed to firmly establish these functional relationships (see also Sect. 6). In the review, we cover both unique and shared EV cargo and discuss implications for biomarker discovery and the design of future therapeutic strategies.

## EV cargo in AD

**Fig.2:**
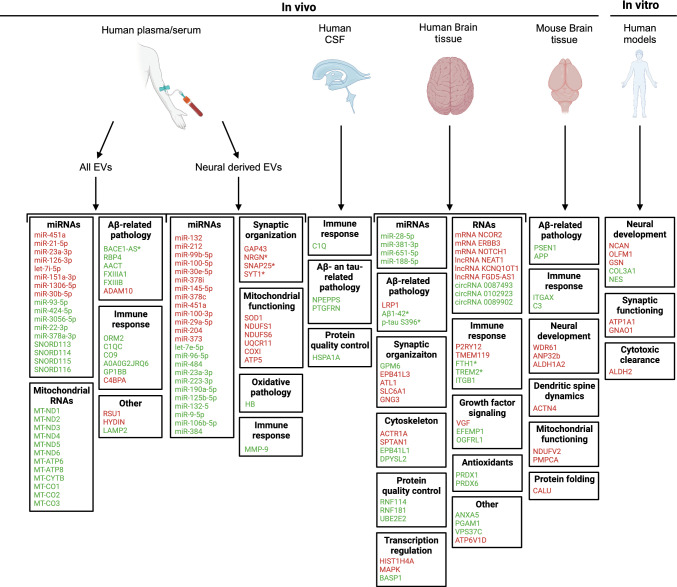
Changes in EV cargo in human AD patients and experimental models. Schematic overview of reported changes in the cargo (RNA or protein) of extracellular vesicles (EVs) derived from different fluids and tissues (serum/plasma, cerebrospinal fluid, and brain tissue), species (human and mouse) and experimental models (in vivo mouse and in vitro culture). Long RNAs (mRNA, long non-coding and circular RNA) and proteins are categorized according to reported functions (e.g. neural development) or link to AD pathology. Green, molecules displaying increased levels in EVs as compared to control. In red, molecules displaying decreased levels. Asterisks indicate molecules that been shown to be fluid biomarkers for AD [141, 142]

AD is an adult-onset neurodegenerative disorder and the most common form of dementia. Worldwide, over 55 million people suffer from dementia of which 60–70% of cases are categorized as AD [18]. Early symptoms include mild cognitive impairment and social withdrawal. A progressive decline in cognition and memory is associated with later stages of AD. Pathological hallmarks of AD consist of amyloid-beta (Aβ) plaques and neurofibrillary tangles, which are linked to neuronal loss [19]. Aβ plaques are caused by the sequential cleavage of the Aβ precursor protein (APP) by β—(BACE1) and α-secretases. Neurofibrillary tangles are caused by aggregation of the hyperphosphorylated form of tau. Because of the importance of Aβ and tau pathology in AD progression, previous studies have examined how these proteins are transported throughout the brain and have shown the importance of EVs in the spreading of Aβ and tau pathology in the adult brain [20, 21]. Here, we will summarize and discuss different types of RNA and protein cargo that have been found in EVs in the context of human and experimental AD. Because of the large number of AD-EV studies published, we here focus on studies since 2019 (Fig. 2; Suppl. Table 1).

### RNA cargo in AD

Most of the work on the RNA content of EVs in AD has focused on non-coding RNAs. Non-coding RNAs can be categorized into small (< 200 nucleotides (nt)) and long non-coding (> 200 nt) RNAs (lncRNAs). microRNAs (miRNAs) and small nucleolar RNAs (snoRNAs) are examples of small non-coding RNAs, and linear lncRNAs and circular RNAs (circRNAs) of lncRNAs. These different non-coding RNA species play important roles in for example gene expression regulation and protein synthesis and their dysregulation may therefore contribute to AD pathogenesis [22].

#### Dysregulation of RNAs in EVs from human plasma and serum samples in AD

Several studies have used plasma- and serum samples from AD patients to investigate differentially expressed miRNAs in EVs (Fig. 2; Suppl. Table 1). For example, one study reported reduced levels of miR-451a, miR-21-5p, miR-23a-3p, miR-126-3p, let-7i-5p, and miR-151a-3p in AD patient plasma-derived EVs compared to controls [23]. The targets of these miRNAs are involved in protein phosphorylation, specifically in the MAPK and SMAD pathway, neurodevelopment, inflammation and APP processing [24]. For example, let-7i-5p can target different proteins linked to APP processing; ADAM10, APPBP2 and APP. APPBP2 and APP are directly involved in Aβ generation. ADAM10 is a well-known β-secretase and cleavage of APP by ADAM10 results in sAPPα [25]. sAPPα is neuroprotective and prevents toxic Aβ formation in the brain. Another study found enhanced levels of the let-7 miRNA family member let-7e-5p in neural-derived AD-NDEVs [26]. Although further work is needed to establish the functional consequences of these changes, these studies highlight EV-derived let-7 miRNAs as interesting targets in AD.

RNA sequencing revealed dysregulation of several other miRNAs in AD-EVs, such as miR-96-5p and miR-484 (increased), and miR-99b-5p, miR-100-5p, miR-30e-5p, miR-378i, miR-145-5p, miR-378c and miR-451a (decreased) [26]. These miRNAs are associated with PI3K-Akt, neurotrophin, toll-like receptor, apoptosis and NF-κB signaling pathways. These pathways are interesting in relation to AD as they are linked to cell survival, apoptosis and the immune response. Another study found reduced levels of miR-132 and miR-212 in AD-NDEVs [27]. Both are involved in neural development and inflammation [28], and miR-132 can reduce Aβ- and tau-pathology and neural cell death [29].

Other work on AD-NDEVs found enhanced miR-125b-5p, miR-132-5, miR-9-5p and miR-106b-5p levels [30]. These miRNAs are all involved in the activation of microglia, resulting in cytokine release and inflammation. In the same study, miR-29a-5p was reduced, which is interesting as previous work showed that reduced miR-29a-5p levels causes neural damage through the activation of pro-inflammatory microglia [31]. Experiments using L1CAM-based immunoaffinity purification of NDEVs also revealed miRNA dysregulation linked to the immune response. Both miR-373 and miR-204 were reduced in AD-NDEVs [32] and previous work showed that these miRNAs play a role in the inflammatory response and apoptosis [33, 34]. RNA sequencing and RT-qPCR on RNA from serum-derived EVs identified increased miR-22-3p and miR-378a-3p, and decreased miR-30b-5p in AD-EVs [35]. Previous work using an AD mouse model showed that miR-22-3p overexpression decreased Aβ−40 and Aβ−42 levels [36]. Moreover, miR-378a-3p is positively correlated with the inflammatory response. Upregulation of miR-22-3p and miR-378a-3p could, therefore, have both neurotoxic and neuroprotective properties in AD by reducing Aβ formation and increasing neuroinflammation, respectively.

In addition to studies linking dysregulated miRNAs in AD-EVs to pro-inflammatory neurotoxic effects, other work suggests neuroprotective roles for miRNA changes found in AD-EVs. For example, increased miR-93-5p, miR-424-5p and miR-3065-5p, and decreased miR-1306-5p in plasma-derived AD-EVs may have protective effects [37]. miR-93-5p and miR-424-5p are involved in the negative regulation of the inflammatory response and angiogenesis, both key processes in AD progression. Aβ-aggregates trigger angiogenesis, which results in increased permeability of the BBB and hypervascularity in brains of AD patients [38]. miRNA-mediated inhibition of angiogenesis may counter these effects.

In addition to miRNAs, other types of RNAs have been found to be dysregulated in AD-EVs (Fig. 2; Suppl. Table 1). For example, the lncRNA BACE1-AS, which plays a role in Aβ-pathology, is enhanced in plasma-derived AD-EVs [39]. Furthermore, various mitochondrial RNAs were more abundant in plasma-derived AD-EVs including ND1-6, ATP6, ATP-8, CYTB and CO1-3 [40]. Mitochondrial dysfunction has been linked to AD and is most likely caused by Aβ-aggregates [41]. It is possible that mitochondrial dysfunction causes increased levels of fragmented and damaged mitochondrial material, which results in packaging of this material in EVs to prevent cells from further oxidative stress. This process could lead to increased levels of the MT-RNAs found in this study.

Thus, studies assessing the RNA content of serum and plasma-derived EVs in AD have thus far mostly identified changes in non-coding RNAs (miRNAs). Interestingly, many of the pathways linked to these RNA changes are relevant for AD pathogenesis, including APP processing, pro-inflammatory responses, apoptosis and neuroprotection.

#### Dysregulation of RNAs in EVs from human brain tissue

A few studies have examined differentially expressed RNAs in EVs from postmortem brain tissue of AD patients (Fig. 2; Suppl. Table 1). For example, one study assessed EVs derived from the parietal cortex and used CD11b-based immunoaffinity to specifically isolate microglial EVs [42]. Using miRNA expression panels, miR-28-5p, miR-381-3p, miR-651-5p, and miR-188-5p were shown to be increased in microglial-derived AD-EVs. These miRNAs are predominantly linked to pro-inflammatory processes. Other work focused on differentially expressed long RNAs in EVs derived from the frontal cortex of AD patients [43]. RNA sequencing revealed dysregulation of several mRNAs, lncRNAs and circRNAs. For example, NCOR2, ERBB3 and NOTCH1 were decreased. These proteins have roles in axon guidance and ion transport and their possible downregulation could contribute to changes in neural circuitry observed in AD. This idea is supported by the downregulation of several lncRNAs, i.e. NEAT1, KCNQ1OT1 and FGD5-AS1, with roles in neuron spine and cell body homeostasis, as well as ion transport. Similarly, hsa_circ_0087493, hsa_circ_0102923 and hsa_circ_0089902 were found be dysregulated and their host genes are involved in synapse organization. circRNAs form as a result of backsplicing, a process introducing a covalent bond between 5’- and 3’-splice sites in a pre-mRNA molecule. A well-characterized mechanism-of-action of circRNAs is their ability to sequester miRNAs or proteins [44, 45].

Thus, EV-derived miRNAs from human brain tissue that show changes in AD have been implicated in immune responses, as observed for serum-derived EVs (Sect. “Dysregulation of RNAs in EVs from human plasma and serum samples in AD.”). Interestingly, changes in long RNAs in these human brain EVs appear to converge on pathways that control neuronal morphology and synaptic connectivity, both of which are affected in AD.

### Protein cargo in AD

The protein cargo of EVs in AD has been studied and validated in different fluids, tissues and models using mass spectrometry, western blotting and ELISA. Here we discuss these observations for the different samples (Fig. 2; Suppl. Table 1).

#### Dysregulation of proteins in EVs from plasma and serum samples in AD

Several different studies have assessed protein expression in AD-EVs from plasma and serum samples (Fig. 2; Suppl. Table 1). One study found increased FXIIIA1, FXIIIB, ORM2 and RBP4, and decreased HYDIN in plasma-derived AD-EVs [46]. The coagulation factors FXIIIA1 and FXIIIB are involved in crosslinking Aβ into stable multimers that may contribute to the Aβ aggregation associated with AD [47]. In another study, ORM2 was increased and found to be negatively regulating the immune response during neuroinflammation [48]. In addition, it affects blood–brain barrier (BBB) integrity by increasing the transcription of proteins involved in tight junctions [49]. RBP4, a protein that binds Aβ and prevents its oligomerization, is also increased in AD-EVs [50]. In addition to changes that link to AD pathology, analysis of AD-NDEVs revealed downregulation of the synaptic proteins GAP43, NRGN, SNAP25 and SYT1 [51]. This is in line with loss of synaptic connectivity in AD and with changes observed in long RNAs in human brain EVs (Sect. “Dysregulation of RNAs in EVs from human brain tissue.”). In addition, decreased levels of mitochondrial proteins were found, including in SOD1, NDUFS1, NDUFS6, UQCR11, COXI and ATP5 [52]. This dysregulation in mitochondrial proteins is in line with the changes in MT-RNAs discussed in Sect. “Dysregulation of RNAs in EVs from human brain tissue.”. It should be noted that although these observations hint at mitochondrial changes, the EV protein levels were decreased whereas mRNA levels in EVs were increased.

Proteomic profiling of plasma-derived AD-EVs revealed elevated levels of A0A0G2JRQ6, C1QC, CO9, GP1BB and RSU1, and reduced ADAM10 [53]. The complement pathway-associated proteins C1QC and CO9 induce the inflammatory response and upregulation of these proteins could promote AD-associated neuroinflammation. Moreover, the coagulation factor GP1BB has been associated with increased inflammation in ischemic stroke [54]. Another link to inflammation in these data is provided by the reported upregulation of MMP-9, a matrix metalloproteinase with a role in the inflammatory response [55], in plasma-derived NDEVs [56]. Decreased levels of ADAM10 (α-secretase) found in AD-EVs may contribute to Aβ aggregation as ADAM10 cleaves APP into a neuroprotective sAPPα isoform (as discussed for let-7 miRNAs targeting ADAM10 in Sect. “Dysregulation of RNAs in EVs from human plasma and serum samples in AD.”)[25]. Other studies found dysregulation of proteins linked to Aβ aggregation as well. Here, mass spectrometry and ELISA validation of AD-EVs identified increased AACT levels. AACT can promote Aβ aggregation and tau phosphorylation [57]. In the same study, a reduction of C4BPα was found, which negatively regulates the immune response [58, 59].

Thus, analysis of differential protein expression in plasma-derived AD-EVs identifies several proteins linked to AD-relevant pathways such as Aβ aggregation, synaptic connectivity, and inflammation, which is in line with pathways identified based on differential RNA expression (Sect. “RNA cargo in AD.”).

#### Dysregulation of proteins in EVs from cerebrospinal fluid in AD

CSF is a valuable resource for monitoring changes in RNA or protein expression in the brain, as brain tissue is less accessible during life. Not many studies have analysed differential protein expression in EVs in CSF from AD patients (Fig. 2; Suppl. Table 1). One study reported elevated levels of HSPA1A, NPEPPS and PTGFRN when comparing EVs from AD patients to those from patients with mild cognitive impairment [60]. Interestingly, these three proteins have been linked to Aβ and tau pathology [61–63]. Another study found increased levels of the complement protein C1Q in CSF-derived AD-EVs [64]. This is in line with the proteome analysis of plasma-derived EVs, which also revealed upregulation of several complement proteins (Sect. “Dysregulation of proteins in EVs from plasma and serum samples in AD.”).

#### Dysregulation of proteins in EVs from human brain tissue in AD

Multiple studies have investigated AD-EVs derived from postmortem brain tissue (Fig. 1; Suppl. Table 1). For example, AD-EVs were isolated from microglia from the parietal cortex using ultracentrifugation and CD11b-based immunoaffinity [42]. Mass spectrometry revealed downregulation of the homeostatic microglial markers TMEM119 and P2RY12, and upregulation of the pro-inflammatory microglial markers FTH1 and TREM2. In line with this dysregulation in proteins related to inflammatory processes, astrocyte-derived EVs isolated in another study using LRP1- and EAAT1-based immunoaffinity revealed enhanced ITGB1 and decreased LRP1 levels [65]. Interestingly, ITGB1 plays a role in the immune system and LRP1 is associated with Aβ and tau clearance [66, 67]. Thus, the limited data available for human brain EVs identifies differential protein expression related to the immune response and the development of Aβ and tau pathology.

#### Dysregulation of proteins in EVs from mouse brain tissue in AD

Several studies have used the double transgenic APP/PSEN1 mouse model to perform EV profiling (Fig. 2; Suppl. Table 1). One study found increased levels of complement protein C3 in EVs of APP/PSEN1 mice [68]. In line with this, a different study identified an increase in the complement system-associated protein ITGAX in EVs from the brain of APP/PSEN1 mice [69]. Other studies identified differential expression of proteins related to AD pathology, neural connectivity and mitochondrial function, which is in line with observations in EVs isolated from the human AD brain and plasma samples (Sect. “Dysregulation of proteins in EVs from plasma and serum samples in AD.” and “Dysregulation of proteins in EVs from human brain tissue in AD.”).

#### Dysregulation of proteins in EVs from in vitro models of AD

Several in vitro models have been used to study AD-EVs. One study investigated the EV cargo profile of neuronal cultures in which mutant tau expression was induced [70]. This revealed reduced NCAN, OLFM1, ALDH2, ATP1A1, GNAO1, GSN, and increased NES and COL3A1 levels. Most of these proteins have roles in neural development and synaptic functioning, in line with observations in AD-EVs from plasma and postmortem brain samples (Fig. 2; Suppl. Table 1).

Overall, extensive changes in EV cargo have been observed in AD in different sample types for both RNAs and proteins. In general, more differentially expressed RNAs were reported as compared to proteins, which could be related to the different sensitivity of the detection methods. Differentially expressed RNAs in AD-EVs were linked to processes such as neural development, inflammation, cell survival, neural circuitry, mitochondrial dysfunction and APP processing. Similar pathways were linked to differentially expressed proteins. AD-EVs showed reduced levels of proteins involved in neural development and synaptic functioning, and upregulation of proteins with roles in Aβ aggregation, tau phosphorylation, inflammation, and protein quality control. Most of these pathways converge onto processes linked to immune function, AD pathology and synaptic connectivity, which are major hallmarks of AD. Interestingly, changes in EV cargo linked to these pathways were observed in different human samples (serum, CSF and brain tissue) and experimental models (mouse and culture). Mouse and in vitro models may therefore represent valuable tools for studying the cause and consequence of EV cargo changes in AD.

## EV cargo in ALS

**Fig.3:**
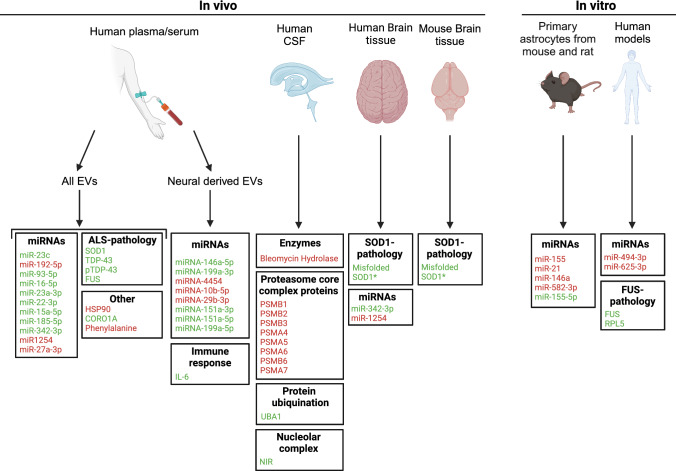
Changes in EV cargo in human ALS patients and experimental models. Schematic overview of reported changes in the cargo (RNA or protein) of extracellular vesicles (EVs) derived from different fluids and tissues (serum/plasma, cerebrospinal fluid, and brain tissue), species (human and mouse) and experimental models (in vivo mouse and in vitro culture). Proteins are categorized according to reported functions (e.g. immune response) or link to ALS pathology. Green, molecules displaying increased levels in EVs as compared to control. In red, molecules displaying decreased levels. Asterisks indicate molecules that have been shown to be pathological proteins associated with ALS [143]

ALS is an adult-onset neurodegenerative disease affecting upper and lower motor neurons causing muscle atrophy. ALS patients have a life expectancy of 2–5 years after diagnosis and death usually occurs due to respiratory failure [2]. ALS arises from a combination of genetic and environmental factors [71, 72] and about 5–10% of ALS cases are classified as familial (f) ALS cases because of demonstration of direct inheritance. The remaining cases are classified as sporadic (s) ALS cases. Currently, mutations in > 40 genes have been reported to explain a large proportion of fALS cases, and genetic defects are also found in 5–17% of sALS patients. For example, SOD1 contains mutations in 12–20% of fALS cases [73]. Normally, this protein has antioxidant properties [74], but when mutated it forms toxic aggregates that induce motor neuron death. Hexanucleotide repeat expansions in C9ORF72 are another and the most frequent genetic cause of ALS [72, 73]. Hexanucleotide repeat expansions cause C9ORF72 haploinsufficiency, development of RNA foci, and the generation of toxic dipeptide repeat proteins [75]. Other common ALS-associated genetic mutations can be found in FUS and TDP-43, both involved in gene expression regulation [71, 72]. FUS is an RNA binding protein and ALS-associated mutations in FUS cause cytoplasmic mislocalization and aggregation of the FUS protein leading to ALS pathology. TDP-43 mutations cause gain- and loss-of-function phenotypes as a result of protein aggregation and mislocalization. Despite extensive research into the pathogenic mechanisms underlying ALS, the number of effective therapies for this disease is still limited. Therefore, the development of new and more effective therapies is urgently needed. Different EV cargo have been studied in the context of ALS, including pathogenic proteins, such as mutant SOD1 or TDP-43, but also other differentially expressed RNAs and proteins. Intercellular transfer of pathogenic proteins via EVs likely contributes to the onset and progression of ALS. Here, we will summarize and discuss different types of RNA and protein cargo that have been found and change in EVs in the context of human and experimental ALS (Fig. 3; Suppl. Table 2).

### RNA cargo in ALS

#### Dysregulation of RNAs in EVs from human plasma and serum samples in ALS

Multiple studies have investigated differentially expressed miRNAs in EVs derived from plasma samples (Fig. 3; Suppl. Table 2). Here, we will discuss miRNAs that were dysregulated and validated. For example, in one study ALS-NDEVs were shown to contain increased levels of miR-146a-5p, miR-199a-3p, miR-151a-3p, miR-151a-5p and miR-199a-5p, and decreased levels of miR-4454, miR-10b-5p and miRNA-29b-3p [76]. In a second study using a larger patient cohort, changes in the levels of miR-4454, miR-10b-5p, miRNA-29b-3p, miR-151a-5p and miR-146a-5p were confirmed by qPCR [77]. miR-146a-5p has been associated with synaptic plasticity and inflammation [78, 79]. For example, it was demonstrated that upregulation of miR-146a-5p increases MAP1B expression, which in turn enhanced AMPA receptor endocytosis causing reduced synaptic transmission [78]. Therefore, it could be argued that elevated levels of miR-146a-5p in ALS-EVs might reduce synaptic plasticity in recipient cells. Increased miR-146a-5p levels in EVs have also been associated with AD. Enhanced expression of this miRNA in the superior temporal lobe neocortex resulted in increased inflammation [79]. The dysregulation of this miRNA in both AD and ALS hints at a more general role in neurodegeneration. Changes in other miRNAs may also be associated with altered synaptic structure and function. For example, increased miR-10b-5p expression leads to lower BDNF protein levels [80]. Therefore, a downregulation of this miRNA, as observed in ALS-EVs, may trigger increase BDNF expression, which in turn could have a positive effect on synaptogenesis and neural survival. miRNAs known to target synapse-related proteins are also frequently increased in ALS-EVs, e.g. miR-23c levels were increased in ALS-EVs [81]. Target gene prediction of miR-23c and gene ontology analysis of the resulting target genes revealed biological processes such as synapse development, synapse assembly and vesicle-mediated transport. Therefore, a dysregulation of miR-23c could contribute to synaptic dysfunction associated with ALS pathology. Interestingly, other members of the miR-23 family also show differential expression in ALS-EVs [82]. Increased levels of miR-93-5p, miR-16-5p, miR-22-3p and miR-23a-3p were found in ALS-EVs and ectopically increased expression of miR-23a-3p in motor neuron-like NSC-34 cells induced apoptosis in vitro.

Finally, Lo et al. isolated EVs from three types of samples: human plasma, spinal cord and frontal cortex. In all samples, miR-342-3p and miR-1254 levels were increased and decreased, respectively [83]. This is an intriguing observation as changes in plasma EVs appear to reflect those in affected brain tissue, suggesting that these miRNAs may serve as potential biomarkers for disease state or progression. miR-342-3p has been associated with prion-based neurodegeneration [84].

Thus far, studies on the RNA content of ALS-EVs from human plasma samples have largely focused on miRNAs. And although the number of identified changes is less as for example compared to AD studies (Sect. “RNA cargo in AD.”), reported EV cargo changes hint at altered synaptic processes and inflammation.

#### Dysregulation of RNAs in EVs from in vitro models of ALS

EVs isolated from cortical astrocyte cultures generated from a rodent model expressing mutant SOD1 (G93A) showed increased miR-155-5p levels and decreased miR-582-3p levels [85] (Fig. 3; Suppl. Table 2). Analysis of the predicted target genes of these miRNAs suggested functions in neurotrophin factor signaling pathways. Treatment of healthy motor neurons with EVs (exosomes) purified from mutant SOD1 astrocytes resulted in decreased neurite length and motor neuron survival. This effect was rescued upon treatment with miR-155-5p antagomirs, to reduce miRNA levels, suggesting a functional contribution of this miRNA in EVs to the observed phenotypes [85]. Another study identified differential levels of inflammatory miRNAs in EVs from astrocyte cultures generated from the spinal cord or cortex of SOD1-G93A transgenic mice. Interestingly, regulation of these miRNAs, e.g. miR-21, miR-155, and miR-146a, in astrocytes was region-specific with miRNAs being downregulated in cortical astrocytes and upregulated in spinal cord astrocytes. Intriguingly, EVs derived from cultured astrocytes from both regions displayed reduced levels of miR-21, miR-155, and miR-146a [86]. This suggests that miRNA levels in EVs may not always reflect the changes that occur in their donor cell. For example, it was proposed that spinal cord astrocytes retain miRNAs to stimulate astrocyte reactivity, explaining why miRNA levels in the astrocyte are enhanced but decreased in the EVs that these cells generate. Analysis of EVs from induced astrocytes (iAstrocytes) from sporadic or SOD1-ALS patients revealed decreased and increased miR-146a levels in different cases. Elevating miR-146a levels in iAstrocytes from patients displaying reduced EV expression of this miRNA restored lysosomal and synaptic pathways in NSC-34 cells incubated with toxic patient iAstrocytes. These data show that miR-146a downregulation in spinal cord astrocytes is an important pathogenic event but also highlight variability in (EV) miRNA levels between patients [87]. As EVs derived from blood samples from ALS patients show increased miR-146a levels (Sect. “Dysregulation of RNAs in EVs from human plasma and serum samples in ALS.”) it will be important to perform studies that include multiple different tissues or cell types to correlate these different miRNA changes. These discrepancies may have several causes as expression of miRNAs is tightly spatiotemporally regulated. For example, in cultured astrocytes from SOD1-G93A mice miR-146a is upregulated at days in vitro (DIV)5 but not DIV13, while miR-155 was only upregulated at DIV15 [86].

Another study generated iAstrocytes from C9-ALS cases and healthy controls and examined the toxicity of EVs generated from these cultured cells on mouse motor neurons. This showed that EV formation and cargo were altered and that C9-ALS EVs induced neuronal network changes and reduced survival. Specifically, reduced miR-494 levels were reported in C9-ALS iAstrocyte EVs and postmortem cortico-spinal tract tissue from sALS patients. miR-494-3p is a negative regulator of SEMA3A, an important protein involved in neural development and axon guidance [88]. Restoration of miR-494-3p expression in iAstrocytes in these co-cultures not only normalized SEMA3A expression in motor neurons but also induced enhanced survival and neurite growth [89].

Together, these studies show changes in the miRNA cargo of ALS-EVs. Many of these dysregulated miRNAs exhibit functions in synaptic development and maintenance and inflammation. Interestingly, these pathways were also associated with changes in AD-EV cargo (Sect. “EV cargo in AD.”), suggesting a broader role in neurodegenerative disease. Several studies have begun to investigate potential functional roles of ALS-EV-associated miRNA changes, also in astrocytes, and identify disease-relevant effects, for example on neuron survival.

### Protein cargo in ALS

#### Dysregulation of proteins in EVs from plasma and serum samples in ALS

In line with the miRNA data discussed above (Sect. “RNA cargo in ALS.”) changes in the protein content of ALS-EVs are linked to the immune response and inflammatory processes (Fig. 3; Suppl. Table 2). For example, increased IL-6 levels were found in astrocyte-enriched ALS-EVs isolated from plasma [90]. This upregulation positively correlated with disease progression in ALS patients. Another study demonstrated that CORO1A levels are increased in ALS-EVs [91]. Overexpression of CORO1A in NSC-34 cells resulted in increased oxidative stress, apoptosis and autophagy. In addition, overexpression hindered the formation of autolysosomes, which could be related to the lysosomal dysfunction implicated in ALS [92]. Other protein changes observed in ALS-EVs link to protein folding and quality control. Decreased levels of HSP90 were found in EVs isolated from plasma of ALS patients and a SOD1 mouse model [93]. This is in line with prior studies in which lower levels of HSP90 were found in astrocytes derived from SOD1 mice and sporadic ALS patients [94, 95]. HSP90 is heavily involved in protein folding and quality control [96] and downregulation of this protein may affect protein folding and cause aggregation of pathological proteins.

Thus, proteins dysregulated in ALS-EVs are involved in inflammation, lysosomal dysfunction and protein quality control. This link to inflammation is in line with dysregulated RNA profiles in ALS-EVs (Sect. “RNA cargo in ALS.”) and dysregulated cargo in AD-EVs (Sect. “EV cargo in AD.”). The suggested roles in lysosomal dysfunction and protein quality control appears to be more distinctive for ALS.

#### Pathological proteins in EVs in ALS

EVs can transfer pathological proteins between cells, thereby facilitating the spread of pathological proteins across different regions of the brain. TDP-43, SOD1 and FUS are known to form inclusions in ALS, and levels of these three proteins are increased in ALS-EVs, specifically in microvesicles [11] (Fig. 3; Suppl. Table 2) This change is not restricted to neurons as muscle cells from sporadic ALS patients also secrete EVs that contain FUS [97]. In this study, it was shown that toxicity of EVs towards healthy motor neurons was dependent on the amount of FUS present in the EVs. Furthermore, muscle cells derived from ALS patients released more EVs compared to those from control individuals. Misfolded and aggregated SOD1 has also been detected in EVs from brain and spinal cord, both in SOD1 mouse models and SOD1-ALS patients [98]. These findings confirm the presence of major pathological proteins in ALS-EVs supporting the possibility that EVs contribute to the spreading of ALS pathology.

Overall, changes in RNA and protein cargo of ALS-EVs have been observed in different sample types. Observations at the RNA level mostly concern miRNA changes, while a few protein changes have been reported, including the presence of pathological proteins such as mutant SOD1. Interestingly, cargo changes can be cell type- and region-dependent, and changes at the transcriptomic level in the cell may not always reflect those in EVs, sometimes because of biological reasons.

## EV cargo in PD

**Fig.4:**
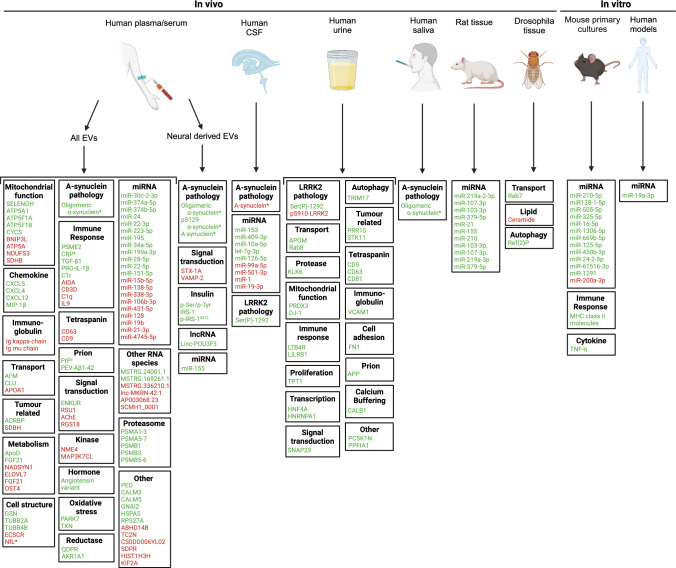
Changes in EV cargo in human PD patients and experimental models. Schematic overview of reported changes in the cargo (RNA or protein) of extracellular vesicles (EVs) derived from different fluids and tissues (serum/plasma, cerebrospinal fluid, urine, saliva, and brain tissue), species (human, rodent and *Drosophila*) and experimental models (in vivo rodent and *Drosophila* and in vitro culture). Proteins are categorized according to reported functions (e.g. immune response) or link to PD pathology. Green, molecules displaying increased levels in EVs as compared to control. In red, molecules displaying decreased levels. Asterisks indicate molecules that have been shown to be fluid biomarkers for PD [144]

PD is the second most common neurodegenerative disorder [99] and affects 8.5 million individuals worldwide [100]. PD is characterized by the progressive loss of dopaminergic neurons in the midbrain and different motor and non-motor symptoms including dyskinesia, dystonia, tremor, sleep disturbances, and cognitive impairment [3, 100, 101]. There is no curative treatment available for PD and the current available therapies focus on alleviating symptoms. PD is categorized into a sporadic (90% of cases) and a familial (10% of cases) form based on patterns of inheritance. Several genetic mutations, such as in *LRRK2* or *SNCA*, have been identified in familial PD [3]. The major molecular pathological hallmark of PD is the presence of Lewy bodies, which are composed of α-synuclein aggregates [102, 103]. Molecular pathways linked to PD are mitochondrial dysfunction, oxidative stress, and inflammation [104–106]. It has been suggested that EVs may serve as a diagnostic tool in PD since brain-derived EVs carry PD associated cargo [107–109]. Here, we will summarize and discuss different types of RNA and protein cargo that have been found and changes in EVs in the context of human and experimental PD (Fig. 4; Supp. Table 3).

### RNA cargo in PD

#### Dysregulation of RNAs in EVs from human plasma and serum samples in PD

Several studies have performed next generation sequencing and qPCR to identify differentially expressed RNAs in serum-derived EVs from PD patients [110–113] (Fig. 4; Supp. Table 3). Pathway analysis of these dysregulated RNAs identify the immune response, cell death, regulation of protein translation and PD-related pathways. Studies have also validated several PD-EV-associated RNAs (mRNA, miRNA, circRNA, antisenseRNA and lncRNA) using qPCR analysis to investigate the use of serum-derived EVs to discriminate between PD patients and healthy individuals. For example, the differential expression of several lncRNAs in PD-EVs, such as lnc-MKRN2-42:1, positively correlated with disease progression [114]. In addition, based on miR-199a-3p, miR- 28-5p, miR-22-5p and miR-151a-5p levels it was possible to distinguish between PD stages II, III and IV [110]. Other miR-22 family members were also shown to be increased in human serum-derived EVs isolated from PD patients [115], suggesting a general role for these miRNAs in PD. miR-128 was decreased in PD-EVs. Overexpressing this miRNA in rat and human in vitro models improved synaptic integrity and prevented 6-OHDA-mediated neuronal apoptosis [116]. Together these results suggest that miRNA changes in PD-EVs may function as disease biomarkers.

#### Dysregulation of RNAs in EVs from cerebrospinal fluid samples in PD

Studies on EVs from CSF are generally sparse in PD but several studies report changes in miRNAs (Fig. 4; Supp. Table 3). One study performed next generation small-RNA sequencing and reported changes in 22 miRNAs in CSF-derived PD-EVs [117]. Three of these miRNAs were validated and could differentiate PD from control samples.

Another study identified 27 differentially expressed miRNAs in PD-EVs and confirmed that miR-1 and miR-19b-3p showed decreased levels and miR-153, miR-409-3p, miR-10a-5p, and let-7 g-3p increased levels [109]. The target genes of these miRNAs are linked to pathways related to dopaminergic synapses, glutamatergic synapses, mTOR signaling and neurotrophin signaling. Decreased levels of miR-19b were also shown in serum-derived PD-EVs [118].

#### Dysregulation of RNAs in EVs from plasma samples of rat models of PD

Whereas EVs isolated from patients can serve as potential biomarkers, experiments in animal models allow the evaluation of molecular changes during the entire disease process. One study used a pre-motor rat model characterized by injections with N-(2-chloroethyl)-N-ethyl-2-bromobenzylamine and 6-OHDA [119] (Fig. 4; Supp. Table 3). Several pro-inflammatory miRNAs were elevated in EVs isolated from plasma of this pre-motor rat model, such as miR-155, miR-21 and miR-210. Interestingly, miR-155 was also increased in NDEVs from plasma of human PD patients. This suggests that the rat model may constitute a translational tool for studying spatiotemporal changes in human miR-155, and perhaps other miRNAs [120].

#### Dysregulation of RNAs in EVs from in vitro models of PD

In vitro models have been used extensively to study PD-EV cargo (Fig. 4; Supp. Table 3). Transgenic SH-SY5Y cells expressing α-synuclein showed increased EV-associated levels of miR-19a-3p [121]. These EVs could enhance the expression of miR-19a-3p in recipient microglia and disrupt autophagy. Interestingly, both miR-19b and miR19b-3p are downregulated in human CSF and serum-derived EV samples in PD [109, 118]. miRNA profiling of dopaminergic cells expressing α-synuclein revealed increased levels of several miRNAs with predicted functions in inflammation, hypoxia, autophagy, and protein aggregation, all of which have been linked to PD [122]. This study also tested the effect of manganese (Mn), which can induce PD. Mn treatment induced an increase in EV release and an altered small RNA profile. Interestingly, miR128-1-5p was increased in Mn-treated cells while miR-128 was decreased in human serum-derived EVs in another study as was discussed before [116]. This discrepancy could indicate cell type-specific changes, differential responses to distinct pathological triggers or technical causes (e.g. differences in sample preparation).

Together, transcriptomic analyses of PD-EVs have largely focused on miRNAs and identify miRNA changes linked to inflammation and synapses. Interestingly, changes in RNA cargo of serum-derived PD-EVs allows discrimination of PD patients and healthy individuals, supporting biomarker potential of PD-EVs.

### Protein cargo in PD

#### Dysregulation of proteins in EVs from human plasma and serum samples in PD

Protein levels in EVs derived from plasma and serum samples have been studied extensively in PD (Fig. 4; Supp. Table 3). Here, we discuss a few studies that provide insight into disease progression mechanism or biomarker potential. Two studies reported increased levels of phosphorylated IRS in PD-EVs and a positive association with the severity of tremor [123, 124]. Further, it was suggested that dysregulated IRS levels may be associated with impaired insulin signalling in PD [125]. Another study showed an increase in PrPc in PD-EVs which correlated with cognitive decline in PD patients [126]. PrPc misfolding causes neurodegenerative prion diseases. Interestingly, several studies on serum-derived PD-EVs identify dysregulated proteins linked to prion disease pathways [127, 128].

Inflammatory proteins are also altered in PD-EVs. Plasma-derived PD-EVs had elevated levels of pro-IL-1β and TGF-β1, both proinflammatory cytokines [129]. Interestingly, PD patients with cognitive deficits had elevated pro-IL-1β, TNF-α, IL-6 and IL-10 levels, but reduced TGF-β1 [129]. Changes in IL-1β, TNF-α, and IL-6 over the course of a year were associated with worsening of motor and non-motor symptoms [130]. Moreover, baseline IL-1β, TNF-α, IL-6, and IL-10 levels were positively associated with motor and non-motor symptoms at follow-up. In addition, another study reported decreased IL-9 levels in PD-EVs and an increase in CRP, MIP-1β, and TNF-α [131]. In the same study, several mitochondrial proteins were shown to be decreased in PD-EVs, including ATP5A, NDUFS3, and SDHB. Interestingly, the neuropeptide VGF has recently been proposed as a neurodegenerative biomarker for PD [132]. Decreased levels of VGF have been identified in CSF, blood, and urine of individuals with AD, ALS, and PD. However, regarding EV cargo decreased levels of VGF were thus far only detected in AD-EVs (Fig. 2; Supp. Table 1). Recent studies have implicated vesicle transport dysfunction in PD pathogenesis, suggesting a possible link to VGF [133]. Therefore, VGF may not only function as a neurodegenerative biomarker, but may also contribute to the onset and/or progression of PD. Overall, changes in the proteome of PD-EVs derived from plasma are associated with impaired insulin signaling, inflammation and mitochondrial dysregulation.

#### Dysregulation of proteins in EVs in human urine samples in PD

Urine samples have also been used to assess the proteome of PD-EVs (Fig. 4; Supp. Table 3). Multiple studies showed elevated pS1292-LRRK levels in urine-derived EVs in PD patients with LRRK2 mutations, which correlated with cognitive impairments [134–136]. In addition, PRDX3, KLK6, TRIM17, TPT1, VCAM1, and LILRB1 levels were increased in urine-derived PD-EVs. These protein changes hint at mitochondrial dysfunction, impaired autophagy, neural cell death and defective neuroinflammation. Proteins related to mitochondrial function and neuroinflammation were also dysregulated in plasma-derived PD-EVs (Sect. “Dysregulation of proteins in EVs from human plasma and serum samples in PD.”).

#### Dysregulation of proteins in EVs from *Drosophila* models of PD.

Mutations in *glucosidase beta acid 1* (*GBA*), which encodes glucocerebrosidase (GCase), are the most penetrant common genetic risk factor for PD and dementia with Lewy bodies and associate with faster disease progression [137]. To investigate how GBA mutations influence pathogenesis, *Drosophila* models of GBA deficiency were created that show neurodegeneration and accelerated protein aggregation (Fig. 4; Supp. Table 3). EVs isolated from GBA-deficient *Drosophila* models show increased protein aggregation, which may promote the spread of pathogenic proteins [138]. In addition, the rescue of GCase deficiency by expression of WT GCase restored GCase levels within the EVs and reduced protein aggregation in EVs, as well as in muscles and brain tissues. These findings support the idea that GCase deficiency induces protein cargo changes in PD-EVs leading to propagation of protein aggregates.

#### Dysregulation of proteins in EVs from in vitro models of PD

The use of in vitro models to study the PD-EV proteome has been limited (Fig. 4; Supp. Table 3). Treatment of the mouse microglia cell line BV-2 with α-synuclein induced enhanced release of EVs with increased levels of TNF-α and MHC class II [139]. This suggests that α-synuclein forces microglia into an activated state. Microglial inflammation is associated with increased apoptosis and α-synuclein-stimulated cells showed increased cell death, which could in part be rescued by neutralization of TNF-α. These results indicate that α-synuclein can act through EV-derived proteins to induce cell death.

Overall, changes in RNA and protein cargo of PD-EVs have been observed in different sample types. RNA changes mostly concern miRNAs and in contrast to AD and ALS a relatively large number of studies have focused on the EV proteome in PD. Together, the reported changes indicate defective mitochondrial function and neuroinflammation. Interestingly, several studies on PD-EVs show correlation between altered EV content and disease parameters, suggesting that EVs are potential sources of biomarkers in PD.

## Conclusion and outlook

In this review, we summarize and discuss recent advances in our understanding of changes in EV cargo in different samples and species or models of three major neurodegenerative diseases. In general, most the reported work focuses on RNA content and protein expression changes are less well studied. This is, however, disease-specific as several proteomic studies have been performed on blood-derived PD-EVs. Similarly, EV studies are more abundant in the field of AD as compared to ALS and PD, and also the nature of subsequent analyses may differ. For example, several studies on ALS-EVs investigate the functional consequences of changes in EV-derived RNA/protein levels, while in PD-EV changes are often correlated to disease parameters. It is important to note that changes in the expression of RNA and protein in EVs may reflect similar changes in the donor cell, although this is not always the case. In addition, changes in EV content may affect recipient cells. In this review, we have attempted to predict the biological process or pathway affected based on (predicted) functions of the RNA or protein. However, much more future work is needed to establish these potential functional consequences or the biomarker potential of EV molecules.

**Fig.5:**
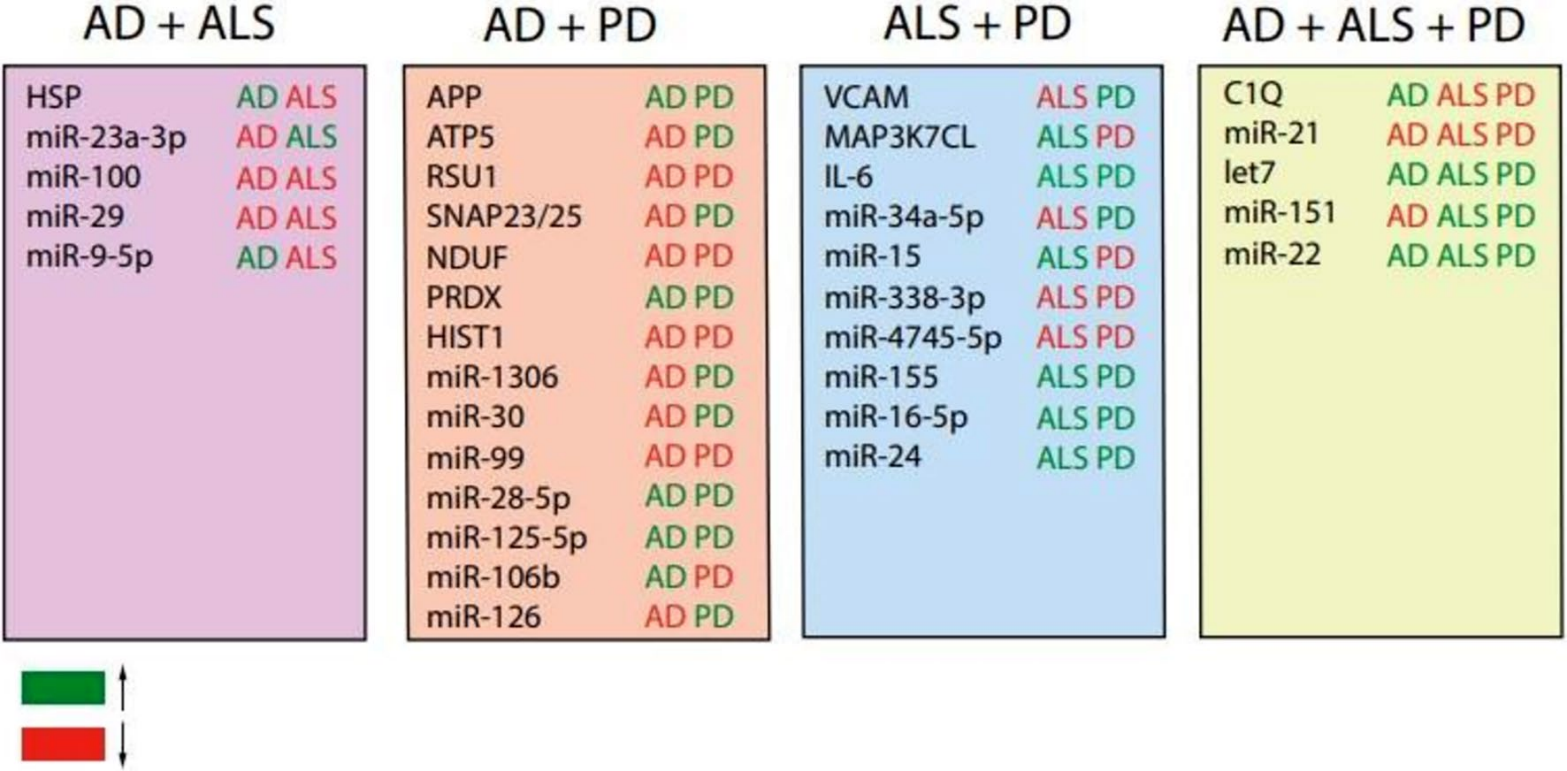
Shared EV cargo changes in neurodegenerative disease. Summary of differentially expressed EV cargo (miRNA or protein) detected in multiple different neurodegenerative diseases (AD, ALS and/or PD). Green, molecules displaying increased levels in EVs as compared to control. In red, molecules displaying decreased levels

**Fig.6:**
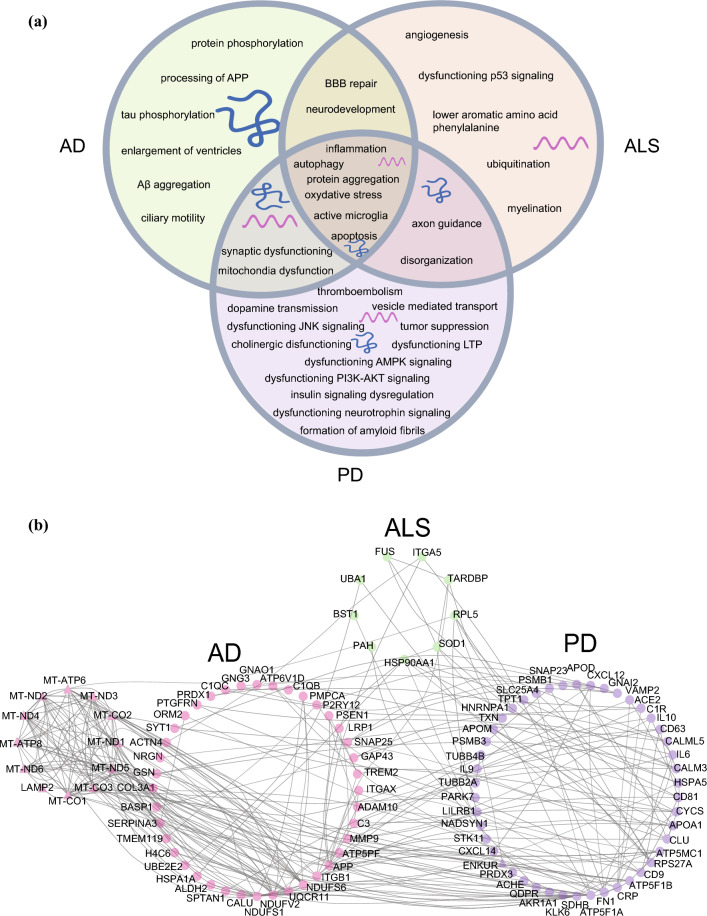
Dysregulated biological processes based on EV cargo changes in neurodegenerative disease. **A** Venn diagram summarizing the affected biological processes predicted based on changes in EV cargo in AD, ALS and/or PD. AD; Alzheimer’s disease, ALS; Amyotrophic lateral sclerosis PD; Parkinson’s disease. **B** Protein–protein interaction network analysis of the dysregulated EV molecules discussed in this review. Cytoscape v3.10.1 software and the String database were used to extract the network of the dysregulated molecules (from Figs. 1, 2 and 3, and Supp. Tables 1, 2 and 3). Triangle shape indicates genes and circular shape indicates proteins. Asterisk indicate proteins dysregulated in EVs in multiple diseases

Interestingly, several of the EV cargo changes discussed are observed in two or all three diseases, although sometimes in opposite directions (Fig. 5). It is likely that these observations highlight shared disease mechanisms (Fig. 6A). For example, decreased levels of the inflammatory miRNA miR-29 were observed in both AD- and ALS derived-EVs [31]. Altered levels of miR-99 and miR-28-5p in AD- and PD-derived EVs and of miR-155 and miR-16-5p in ALS- and PD-EVs also link to inflammation [122, 140]. Interestingly, a few miRNAs were similarly altered in EVs in all three diseases, let7, miR-21 and miR-22, which suggests that these changes may associate with general neurodegenerative disease pathways. miR-151 was also altered in AD, ALS and PD, although in different directions. It is possible that miR-151 is an essential miRNA and that both increased and decreased levels of this miRNA cause pathogenic changes. To more objectively identify pathways downstream of changes in EV-derived RNAs and proteins in AD, ALS and PD, we performed a protein–protein-interaction network (PPIN) analysis (Fig. 6B). Cytoscape v3.10.1 was used with String database to extract the network of the dysregulated molecules (Figs. 1, 2 and 3, and Supp. Tables 1, 2 and 3). This analysis resulted in a network with 107 nodes and 262 edges (PPI score > 0.85) between cargo (changes) of AD-, ALS- and PD-derived EVs. The larger networks for AD- and PD-EVs compared to ALS-EVs reflects the number of studies performed in the respective fields. Highly interconnected molecules and their pathways in this analysis may serve as interesting potential targets for developing therapies. Together, the results discussed in the review highlight abundant changes in the cargo of EVs in neurodegenerative diseases and begin to show the functional effects of these changes. These effects can be cell type-dependent and are also spatiotemporally regulated. In several cases changes in EV cargo are indicative of disease states or able to discriminate disease from healthy control, highlighting the biomarker potential of EV cargo (changes). These are excellent starting points for further developing EVs as sources of biomarkers and for interfering with cargo changes as a means of novel therapies.

## Supplementary Information

Below is the link to the electronic supplementary material.

Supplementary file 1.

## Data Availability

Data generated in this study is avaliable upon request from the corresponding author.
