# Supplementary material for: Shared and distinct changes in the molecular cargo of extracellular vesicles in different neurodegenerative diseases
Source: Cell Mol Life Sci. 2024 Dec 3;81(1):479. doi: 10.1007/s00018-024-05522-7 (PMC11615177; doi:10.1007/s00018-024-05522-7)
Supplement: Supplementary file 1 [file 18_2024_5522_MOESM1_ESM.pdf]

Shared and distinct changes in the molecular cargo of extracellular vesicles in different neurodegenerative diseases.

Cellular and Molecular Life Sciences

Anna F. Wiersema[1], Alyssa Rennenberg[1], Grace Smith[1], Suzy Varderidou-Minasian[1] , and R. Jeroen Pasterkamp[1]

[1] Department of Translational Neuroscience, University Medical Center Brain Center, Utrecht University, Utrecht, The Netherlands

R. Jeroen Pasterkamp. Email: r.j.pasterkamp@umcutrecht.nl.

Supplementary Table 1. Overview of studies on EV cargo in AD.

Model	EV origin	Cell type	EV isolation method	Literature	Method used to determine EV content	Major findings
In vivo	Human	Cortical gray matter	Ultracentrifugation	[10]	ELISA and mass spectrometry	AD-EVs contained elevated levels of pathogenic markers Aβ1-42, p-tau S396, APOE and SNCA. ANXA5 and GPM6A were increased whereas VGF and ACTR1A were decreased in AD-EVs.
In vivo	Human	Plasma	Size-exclusion chromatography (SEC)	[23]	Next-generation sequencing and qPCR	AD-EVS showed increased levels of miR-451a, miR-21-5p, miR-23a-3p, miR-126-3p, let-7i-5p, and miR-151a-3p.
In vivo	Human	Plasma	Ultracentrifugation and L1CAM-based immunoaffinity	[27]	miRNA qPCR	AD neural exosomes revealed decreased levels of miR-132 and miR-212.
In vivo	Human	Microglia-enriched EVs from parietal cortex	Filter purification, ultracentrifugation and CD11b-based immunoaffinity	[42]	Mass spectrometry, western blotting and miRNA expression panels	In microglial AD-EVs homeostatic microglial markers P2RY12 and TMEM119 levels were lower and activated microglial markers FTH1 and TREM2 higher. miR-28-5p, miR-381-3p, miR-651-5p and miR-188-5p levels were increased.
In vivo	Human	CSF	Commercial exosome isolation kit	[60]	Mass spectrometry	AD-EVs showed increased levels of HSPA1A, NPEPPS and PTGFRN compared to EVs isolated from patients with MCI.
In vitro	Human	Astrocyte-enriched EVs from frontal cortical grey matter	Ultracentrifugation and size exclusion chromatography	[65]	Label-free quantitative LC-MS/MS proteomics and western blotting	ITGB1 was increased and LRP1 decreased in astrocyte AD-EVs.
In vivo	Human	Plasma-derived neural EVs	Commercial exosome isolation kit and L1CAM-based immunoaffinity	[140]	LC-MS/MS and ELISA	HB levels are elevated in AD exosomes.
In vivo	Human	Brodmann areas	Ultracentrifugation and size exclusion chromatography	[141]	Mass spectrometry	Antioxidant enzymes PRDX1 and PRDX6 were increased in AD-EVs.
In vivo	Human	Plasma	Commercial exosome isolation kit	[40]	RNA sequencing and RT-qPCR	MT-ND1-6 mRNAs, MT-ATP6 and -8, MT-CYTB and MT-CO1-3 were increased in AD-EVs.
In vivo	Human	Plasma	Commercial exosome isolation kit and L1CAM-based immunoaffinity	[26]	RNA sequencing and RT-qPCR.	let-7e-5p, miR-96-5p and miR-484 levels were increased and miR-99b-5p, miR-100-5p, miR-30e-5p, miR-378i, miR-145-5p, miR-378c and miR-451a were decreased in AD-EVs.

In vivo	Human	Plasma	Commercial exosome isolation kit	[39]	RT-qPCR	lncRNA BACE1-AS was increased in AD-EVs.
In vivo	Human	CSF	Ultracentrifugation and Vn96-based immunoaffinity	[64]	Mass spectrometry and ELISA	Complement protein C1q was increased in AD-EVs.
In vivo	Human	Plasma	Commercial exosome isolation kit and L1CAM-based immunoaffinity	[142]	qPCR	miR-23a-3p, miR-223-3p, and miR-190a-5p levels were increased and miR-100-3p levels decreased in AD-EVs.
In vivo	Human	Plasma	Ultracentrifugation	[46]	LC-MS/MS	FXIIIA1, FXIIIB, ORM2 and RBP4 were increased and HYDIN decreased in AD-EVs.
In vivo	Human	Plasma	Commercial exosome isolation kit and L1CAM-based immunoaffinity	[56]	ELISA	Inflammatory biomarker MMP-9 was increased in AD-EVs.
In vivo	Human	Plasma	Commercial exosome isolation kit and NCAM-based immunoaffinity	[51]	ELISA	GAP43, NRGN, SNAP25, and SYT1 levels were lower in AD-EVs.
In vivo	Human	Plasma	Commercial exosome isolation kit and L1CAM-based immunoaffinity	[30]	qPCR	miR-125b-5p, miR-132-5, miR-9-5p and miR-106b-5p levels were increased and miR-29a-5p decreased in AD-EVs.
In vivo	Human	Hippocampus and prefrontal cortex	Ultracentrifugation	[143]	Mass spectrometry	EPB41L1, BASP1, DPYSL2, PGAM1 levels were higher and SPTAN1, HIST1H4A, EPB41L3, ATP6V1D, ATL1, SLC6A1, GNG3, MAPK lower in AD-EVs.
In vivo	APP/PSEN1 Mouse	Brain tissue	Ultracentrifugation	[69]	Tandem mass tag-mass spectrometry	PSEN1, APP, and ITGAX levels were increased and WDR61, PMPCA, ALDH1A2, CALU, ANP32B, ACTN4, and NDUFV2 decreased in AD-EVs.
In vivo	Human	Plasma	Ultracentrifugation	[144]	RNA sequencing	Small nucleolar RNAs SNORD113, SNORD114, SNORD115 and SNORD116 levels were higher in AD-EVs.
In vivo	Human	Plasma	Commercial exosome isolation kit and L1CAM-based immunoaffinity	[52]	ELISA	AD-EVs showed lower levels of mitochondrial complexes SOD1, NDUFS1, NDUFS6, UQCR11, COXI and ATP5.
In vivo	Human	Plasma	Ultracentrifugation	[53]	Mass spectrometry	A0A0G2JRQ6, C1QC, CO9, GP1BB, RSU1 levels were increased and ADAM10 was decreased in AD-EVs.
In vivo	Human	Frontal cortex	Size exclusion chromatography	[43]	RNA sequencing and RT-qPCR	NCOR2, ERBB3 and NOTCH1 levels were decreased, lncRNAs NEAT1, KCNQ1OT1 and FGD5-AS1 were decreased and circRNAs 0087493, 0102923 and 0089902 were increased in AD-EVs.
In vivo	Human	Plasma	Commercial exosome isolation kit	[37]	qPCR	miR-93-5p, miR-424-5p and miR-3065-5p were increased and miR-1306-5p decreased in AD-EVs.
In vivo	Human	Serum	Commercial exosome isolation kits	[57]	Mass spectrometry, ELISA and WB	AACT was increased and C4BPα decreased in AD-EVs.
In vivo	APP/PSEN1 mouse	Choroid plexus	Size exclusion chromatography	[68]	Mass spectrometry	Pro-inflammatory complement protein C3 was increased in AD-EVs.
In vivo	Human	Brodmann area 9	Protein Organic Solvent Precipitation (PROSPR)	[145]	LC-MS/MS	EFEMP1, OGFRL1, RNF114, RNF181, UBE2E2 and VPS37C levels were increased in early AD-EVs.

In vivo	Human	Serum	Commercial exosome isolation kit and L1CAM-based immunoaffinity	[32]	qPCR	miR-204 and miR-373 were decreased in AD-EVs.
In vitro	Human	mTau iPSC-derived neurons	Commercial exosome isolation kit	[70]	LC-MS/MS	NCAN, OLFM1, ALDH2, ATP1A1, GNAO1 and GSN were decreased and NES and COL3A1 increased in mTau EVs.
In vitro	Human	Plasma	Ultracentrifugation and NCAM (and ABCA1)-based immunoaffinity	[146]	qPCR	miR-384 levels were higher in NCAM- and NCAM/ABCA1-labeled AD exosomes.
In vivo	Human	Serum	Commercial exosome isolation kit	[35]	RNA sequencing and qPCR	miR-30b-5p was decreased and miR-22-3p and miR-378a-3p were increased in AD-EVs.
In vivo	Human	Plasma	Commercial exosome isolation kit	[147]	ELISA	LAMP-2 was increased in AD-EVs.

Supplementary Table 2. Overview of studies on EV cargo in ALS.

Model	EV origin	Cell type	EV isolation method	Literature	Method used to determine EV content	Major findings
In vivo	Human	CSF	Ultrafiltration liquid chromatography	[148]	Liquid chromato-graphy-tandem mass spectrometry	ALS-EVs showed decreased levels of bleomycin hydrolase enzyme and proteasome core complex proteins. C9-ALS EVs specifically showed increased levels of UBA1.
In vivo	Human	Plasma	Exoquick precipitation kit, centrifugation and L1CAM-based immunoaffinity	[76]	Next-generation sequencing	ALS-EVs showed increased levels of miR-146a-5p, miR-199a-3p, miR-151a-3p, miR-151a-5p, miR-199a-5p and decreased levels of miR-4454, miR-10b-5p, miRNA-29b-3p levels.
In vivo	Human	Plasma	Polymer based precipitation and L1CAM-based immunoaffinity	[77]	qPCR	Sporadic ALS-EVs showed decreased levels of miRNA-4454, miRNA-10b-5p and miRNA-29b-3p and increased levels of miRNA-151a-5p and miRNA-146a-5p.
In vivo	Human	Plasma	Polyethylene glycol, centrifugation and L1CAM-based immunoaffinity	[149]	ELISA	The levels of HERV-K in neural EVs of advanced-phase ALS patients were higher when compared to early-phase ALS patients. However, no significant difference was found in HERV-K EV levels between healthy control and ALS patients.
In vivo	Human	Plasma	VN96-precipitation and centrifugation	[150]	Next-generation sequencing and droplet digital PCR	miR-15a-5p and miR-185-5p levels were increased in ALS-EVs.
In vitro	Mouse	Spinal and cortical astrocytes	Ultracentrifugation and filtering	[86]	qPCR	miR-155, miR-21 and miR-146a were decreased in ALS-EVs.
In vitro	Human	Astrocytes	Ultracentrifugation and filtration	[89]	Microarray analysis	miR-494-3p was decreased in C9ALS-EVs.
In vivo	Human	Plasma	Differential ultracentrifugation and filtration	[11]	Western Blot	ALS-MVs contained higher SOD1, TDP-43, phospho-TDP-43, and FUS levels.
In vivo	Human and mouse	Plasma	Ultracentrifugation in parallel with NBI (nickel-based isolation)	[93]	Western blot and mass spectrometry	Decreased levels of HSP90 were found in ALS-EVs.
In vitro	Human	Muscle cells	Differential ultracentrifugation and filtration	[97]	Mass spectrometry and western blot	ALS-EVs were enriched in FUS protein and its binding partner RPL5 and ALS muscle cells produced more EVs than control EVs.
In vivo	Human and mouse	Primary neural cell culture	Centrifugation	[98]	Quantitative proteomics, immuno-precipitation and ELISA	Both EVs from brain and spinal cord of SOD1-mice and human SOD1 patients contained misfolded and nonnative disulfide-cross-linked aggregated SOD1.
In vivo	Human	Plasma	Exoquick precipitation kit and ACSA-1 immunoprecipitation	[90]	ELISA	IL-6 levels of astrocyte-derived ALS-EVs were increased.
In vivo	Human	Plasma	Exosome isolation kit	[91]	Mass spectrometry and western blot	CORO1A levels were increased in ALS-EVs.
In vivo	Human	Plasma, spinal cord and frontal cortex	Immuno-based affinity (exochip)	[83]	NanoString miRNA profiling	In all 3 sources of tissue miR-342-3p was increased and miR-1254 was decreased in ALS-EVs.
In vitro	Human	Motor neurons	Ultracentrifugation	[151]	TaqMan® Low Density Arrays and qPCr	miR-625 -3p was decreased in C9-ALS-EVs but increased in TARDBP-ALS-EVs.
In vivo	Human	Plasma	Exoquick and exoEasy Maxi Kit	[152]	qPCR	Decreased levels of miR-27a-3p in ALS-EVs.
In vivo	Human	CSF	Gel filtration chromatography	[153]	Liquid chromato-graphy-tandem mass spectrometry	Nucleolar complex protein 2 homolog (NIR) was enhanced in ALS-EVs.

In vivo	Human	Plasma	ExoEasy Maxi Kit	[81]	Small RNA sequencing and digital droplet PCR (ddPCR)	miR-23c levels were increased and miR-192-5p decreased in ALS-EVs.
In vivo	Human	Plasma	ExoEasy Maxi Kit	[82]	Small RNA sequencing and qPCR	miR-93-5p, miR-16-5p, miR-23a-3p and miR-22-3p were increased in ALS-EVs.
In vitro	Rat	Cortical astrocytes	Ultracentrifugation	[85]	qPCR	miR-155-5p levels were increased and miR-582-3p decreased in ALS-EVs.

Supplementary Table 3. Overview of studies on EV cargo in PD.

Model	EV origin	Cell type	EV isolation method	Literature	Method used to determine EV content	Major findings
In vivo	Human	Plasma	ExoQuick kit	[127]	ELISA, western blot	PD-EV prion protein concentration was increased.
In vivo	Human	Plasma	Centrifugation and size exclusion chromatography	[154]	2D differential gel electrophoresis	CLU, C1R and APOA1 were decreased in PD-EVs.
In vivo	Human	Urine	Centrifugation	[155]	Mass spectrometry and quantitative immunoblots	Calbindin and SNAP23 were increased in PD-EVs.
In vivo	Human	Urine	Centrifugation and filtration	[156]	Western blot	DJ-1 levels were increased in PD-EVs and increased in an age-dependent manner in male PD patients.
In vivo	Human	Urine and CSF	Centrifugation	[135]	Mass spectroscopy	Urinary pS1292- LRRK2 levels were increased in PD-EVs of male patients carrying a LRRK2 mutation.
In vivo	Human	CSF	Nanoparticle tracking analysis	[118]	Next-generation small-RNA sequencing	Signature of increased miR-126-5p and decreased miR-99a-5p and miR-501-3p in EVs can differentiate PD from control samples.
In vivo	Human	Plasma-derived erythrocyte s	Centrifugation and filtration	[157]	NanoLC/MSMS	AIDA, ABHD14B, NADSYN1, QDPR and CNRIP1 were decreased in PD-EVs. AKR1A1, ATP5A1 and USP24 were increased in PD-EVs.
In vivo	Rat	Midbrain and plasma	Ultracentrifugation, SEC and ultrafiltration	[158]	Next-generation sequencing	miR-103-3p, miR-107-3p, miR-219a-3p and miR-379-5p were increased in PD-EVs.
In vivo	Human	Plasma	Centrifugation and ExoEasy Maxi kit	[130]	Western Blot	pro-IL- 1β and TNF-α in were increased in PD-EVs. EVs isolated from PD patients with cognitive deficits also showed higher pro-IL-1 β, IL-6, TNF-α, IL-10, and lower TGF-β1 levels compared to PD without cognitive deficits.
In vivo	Human	Plasma	Centrifugation, exoEasy Maxi Kit	[131]	Western blot	IL-1β and TGF-β1 levels were increased in PD-EVs.
In vivo	Human	Plasma	ExoQuick Kit and L1CAM-based immunoaffinity	[125]	ELISA	Ratio of p-Ser/p- Tyr IRS-1 was higher in PD-EVs.
In vivo	Human	Plasma	ExoEasy Maxi Kit	[159]	qPCR	miR-30c-2-3p was higher and miR-15b-5p, miR-138-5p, miR-338-3p, miR-106b-3p and miR-431- 5p lower in PD-EVs.
In vivo	Human	Serum	Ultracentrifugation	[111]	qPCR	miR-374a-5p and miR-374b- 5p levels were increased in PD-EVs. Decreased miR-199a-3p levels in PD-EVs distinguished stage II, increased miR-28-5p distinguished stage III and miR-22-5p and miR-151a-5p distinguished stage IV.
In vivo	Human	Plasma	Immunoprecipitatio n	[160]	Western blot	p-IRS-1^{S312} was increased in PD-EVs.
In vivo	Human	Urine	Ultracentifugation	[134]	Western blot	pS1292-LRRK2 levels were increased in humans with LRRK2 G2019S mutation. Rab8 was higher and pS910-LKKR2 and S935-LRRK2 lower in PD-EVs.
In vivo	Human	Urine	Chemical affinity, EV trap and ultracentrifugation	[161]	LC-MS, qPCR, parallel reaction monitoring (PRM), western blot	PRDX3, KLK6, TRIM17, TPT1, VCAM1, LILRB1, PCSK1N, HNRNPA1, LTB4R, PLA2G4A, PRR15, PPFIA1, HNF4A, FN1, APP, APOM, STK11, CD9, CD63 and CD81 levels were increased in PD-EVs.
In vivo	Human	Plasma	Centrifugation and immunoprecipitatio n	[162]	ELISA	pS129-α-syn was increased in PD-EVs and NfL decreased in female PD-EVs.

In vivo	Human	Serum	Ultracentrifugation and filtration	[132]	Multiplex immunoassay	CD9, CD63, IL9, FGF21, ATP5A, NUDFS3 and SDHB were decreased in PD-EVs. CRP, TNF-α and MIP-1β were increased in PD-EVs.
In vivo	Human	Plasma	SEC and L1CAM-based immunoaffinity	[121]	LC-MS	ATP5F1A, ATP5F1B, CALM3, CALML5, CYCS, GNAI2, HSPA5, PARK7, PSMA1-3, PSMA5-7, PSMB1, PSMB3, PSMB5-6, RPS27A, SLC25A4, TUBB2A, TUBB4B, TXN were increased in PD-EVs.
In vivo	Human	Plasma	Differential centrifugation and filtration	[113]	Next-generation sequencing	mRNAs for AP003068.23, MAP3K7CL, RSU1, ELOVL7, RGS18, TC2N, HIST1H3H, SDPR, BNIP3L, OST4, and KIF2A were decreased in PD-EVs whereas mRNAs for PSME2 and SELENOH was increased in PD-EVs.
In vivo	Human	CSF	Centrifugation	[110]	Taqman miRNA arrays, qPCR	miR-153, miR-409-3p, miR-10a-5p, and let-7g-3p levels were increased and miR-1 and miR-19b-3p decreased in PD-EVs.
In vivo	Human	Plasma	Centrifugation, ultracentrifugation	[163]	Nano-scale flow cytometry	The ratio of Aβ1-42 in PD-EVs was increased. Also PD-EVs from patients with dementia had higher Aβ1-42 ratio compared to non-dementia PD.
In vivo	Human	Plasma	Ultracentrifugation, density gradient	[164]	qPCR	miR-34a-5p was increased in PD-EVs.
In vivo	Human	Serum	Centrifugation	[165]	Western blot, qPCR	miR-3147, miR-4497, miR-1915-3p, miR-3960, miR-1665, miR-4787-5p, miR-2861, miR-4488, miR-4745-5p, miR-4466, miR-1469, miR-3940-5p were decreased in PD-EVs. miR-4485-3p and miR-320c were increased in PD-EVs.
In vitro	Mouse	Cell culture	Differential centrifugation and filtration	[123]	Next-generation sequencing	miR-210-5p, miR128-1-5p, miR-505-5p, miR-325-5p, miR-16-5p, miR-1306-5p, miR-669b-5p, miR-125-5p, miR-450b-3p, miR-24-2-5p, miR-61516-3p and miR-1291 were increased in PD-EVs.
In vivo	Drosophila	Whole body	Centrifugation and filtration	[137]	LC-MS	Ref(2)P (Drosophila homologue of mammalian p62) and Rab7 levels were increased and ceramide decreased in PD-EVs.
In vivo	Human	Plasma	Ultracentrifugation	[166]	ELISA	AChE was decreased in PD-EVs and correlated with disease severity.
In vivo	Human	Plasma	Ultracentrifugation and filtration	[114]	Next-generation sequencing and qPCR	SCMH1_0001 and its target genes ARID1 and C1orf115 were decreased in PD-EVs
In vivo	Human	Plasma	miRCURY exosome serum/plasma kit, centrifugation	[117]	qPCR	miR-128 levels were decreased in PD-EVs.
In vivo	Human	Serum	Centrifugation, total exosome isolation reagent	[119]	qPCR	miR-19b was decreased and miR-195 and miR-24 increased in PD-EVs.
In vivo	Rat	Plasma	Differential centrifugation	[120]	Spectro-photometer, qPCR, LC-MS	miR21, miR155 and miR210 were increased in PD-EVs.
In vitro	Human	SH-SY5Y cells	Centrifugation	[122]	MiRCURY Array, qPCR	miR-19a-3p was increased in EVs from SNCA-positive cells.
In vivo	Human	Urine	Centrifugation	[133]	Immuno-blotting	pS-1292 LRRK2 levels were increased in idiopathic PD-EVs.
In vivo	Human	Serum	Centrifugation, ExoQuick exosome precipitation solution kit	[116]	qPCR	miR-21-3p was decreased and miR-22-3p and miR-223-5p were increased in PD-EVs.
In vitro	Mouse	Microglia cell line BV-2 cells	Centrifugation and ultracentrifugation	[138]	Western blot, flow cytometry	Surface expression levels of MHC class II molecules and surface TNF-α was increased in PD-EVs.

In vitro	Mouse	Astrocytes	Ultracentrifugation and filtration	[167]	Small- RNA sequencing, qPCR	miR-200a-3p levels were decreased in EVs isolated from MPP$^+$-stimulated astrocytes.
In vivo	Human	Serum	Centrifugation, ultracentrifugation	[129]	Mass spectrometry	CLU, C1q, AFM, AGT, ApoD, GSN and PEDF progressively increased in EVs from mild to severe PD. Levels of human neuroblastoma full-length cDNA clone CS0DD006YL02, precusor (AA-19 to 113), C1q, mysion-reactive immuno-globulin kappa chain, Ig kappa chain V-III region, immunoglobulin mu-chain and immunoglobulin kappa variable 1 to 33 progressively decreased in EVs from mild to severe PD.
In vivo	Human	Plasma	Ultracentrifugation	[115]	Next-generation sequencingqPCR	lncRNAs MSTRG.24001.1 and MSTRG.169261.1; mRNAs ACRBP, CXCL5, and ENKUR were increased in PD-EVs. lncRNAs MSTRG.336210.1 and lnc-MKRN2-42:1; and mRNAs NME4, CD3D, and ECSCR were decreased in PD-EVs.
In vivo	Human	Plasma	Commercial kit (UR52136, Umibio)	[168]	MALDI-TOF MS and electron multiplier (EM)	CXCL12 related peptides and CXCL4 protein levels were increased in PD-EVs.
In vivo	Human	Plasma	Magnetic microbeads, centrifugation and L1CAM-based immunoaffinity	[169]	qPCR	Linc-POU3F3 and SNCA levels were increased in PD-EVs.
In vivo	Human	Serum	ExoQuick Kit and L1CAM-based immunoaffinity	[170]	Western blot	Oligomeric SNCA levels were increased and STX-1A and VAMP-2 were decreased in PD-EVs.

Additional references

[146] B.I. Arioz, K.U. Tufekci, M. Olcum, D.Y. Durur, B.A. Akarlar, N. Ozlu, H.A. Bagriyanik, P. Keskinoglu, G. Yener, S. Genc, Proteome profiling of neuron-derived exosomes in Alzheimer’s disease reveals hemoglobin as a potential biomarker, Neurosci Lett 755 (2021) 135914. https://doi.org/10.1016/j.neulet.2021.135914.

[147] Y. Huang, T.A.P. Driedonks, L. Cheng, H. Rajapaksha, D.A. Routenberg, R. Nagaraj, J. Redding, T. Arab, B.H. Powell, O. Pletniková, J.C. Troncoso, L. Zheng, A.F. Hill, V. Mahairaki, K.W. Witwer, Brain Tissue-Derived Extracellular Vesicles in Alzheimer’s Disease Display Altered Key Protein Levels Including Cell Type-Specific Markers, Journal of Alzheimer’s Disease 90 (2022) 1057–1072. https://doi.org/10.3233/JAD-220322.

[148] M. Serpente, C. Fenoglio, M. D’Anca, M. Arcaro, F. Sorrentino, C. Visconte, A. Arighi, G.G. Fumagalli, L. Porretti, A. Cattaneo, M. Ciani, R. Zanardini, L. Benussi, R. Ghidoni, E. Scarpini, D. Galimberti, MiRNA Profiling in Plasma Neural-Derived Small Extracellular Vesicles from Patients with Alzheimer’s Disease, Cells 9 (2020) 1443. https://doi.org/10.3390/cells9061443.

[149] V. Bodart-Santos, L.S. Pinheiro, A.J. da Silva-Junior, R.L. Froza, R. Ahrens, R.A. Gonçalves, M.M. Andrade, Y. Chen, C. de L. Alcantara, L.T. Grinberg, R.E.P. Leite, S.T. Ferreira, P.E. Fraser, F.G. De Felice, Alzheimer’s disease brain-derived extracellular vesicles reveal altered synapse-related proteome and induce cognitive impairment in mice, Alzheimer’s & Dementia 19 (2023) 5418–5436. https://doi.org/10.1002/alz.13134.

[150] N.F. Fitz, J. Wang, M.I. Kamboh, R. Koldamova, I. Lefterov, Small nucleolar RNAs in plasma extracellular vesicles and their discriminatory power as diagnostic biomarkers of Alzheimer’s disease, Neurobiol Dis 159 (2021) 105481. https://doi.org/10.1016/j.nbd.2021.105481.

[151] X. Gallart-Palau, A. Serra, Y. Hase, C.F. Tan, C.P. Chen, R.N. Kalaria, S.K. Sze, Brain-derived and circulating vesicle profiles indicate neurovascular unit dysfunction in early Alzheimer’s disease, Brain Pathology 29 (2019) 593–605. https://doi.org/10.1111/bpa.12699.

[152] Y. Li, S. Meng, W. Di, M. Xia, L. Dong, Y. Zhao, S. Ling, J. He, X. Xue, X. Chen, C. Liu, Amyloid-β protein and MicroRNA-384 in NCAM-Labeled exosomes from peripheral blood are potential diagnostic markers for Alzheimer’s disease, CNS Neurosci Ther 28 (2022) 1093–1107. https://doi.org/10.1111/cns.13846.

[153] G. Krishna, A. KN, R.S. Kumar, B.C. Sagar, M. Philip, A.B. Dahale, T.G. Issac, S.S.R. Mukku, P.T. Sivakumar, S. Subramanian, Higher levels of lysosomal associated membrane protein-2 (LAMP-2) in plasma exosomes from Alzheimer's disease: An exploratory study from South India, Asian J Psychiatr 48 (2020) 101898. https://doi.org/10.1016/j.ajp.2019.101898.

[154] A.G. Thompson, E. Gray, I. Mäger, M.-L. Thézénas, P.D. Charles, K. Talbot, R. Fischer, B.M. Kessler, M. Wood, M.R. Turner, CSF extracellular vesicle proteomics demonstrates altered protein homeostasis in amyotrophic lateral sclerosis, Clin Proteomics 17 (2020) 31. https://doi.org/10.1186/s12014-020-09294-7.

[155] Y. Li, Y. Chen, N. Zhang, D. Fan, Human endogenous retrovirus K (HERV-K) env in neuronal extracellular vesicles: a new biomarker of motor neuron disease, Amyotroph Lateral Scler Frontotemporal Degener 23 (2022) 100–107. https://doi.org/10.1080/21678421.2021.1936061.

[156] D. Saucier, G. Wajnberg, J. Roy, A.-P. Beauregard, S. Chacko, N. Crapoulet, S. Fournier, A. Ghosh, S.M. Lewis, A. Marrero, C. O'Connell, R.J. Ouellette, P.J. Morin, Identification of a circulating miRNA signature in extracellular vesicles collected from amyotrophic lateral sclerosis patients, Brain Res 1708 (2019) 100–108. https://doi.org/10.1016/j.brainres.2018.12.016.

[157] M. Rizzuti, V. Melzi, D. Gagliardi, D. Resnati, M. Meneri, L. Dioni, P. Masrori, N. Hersmus, K. Poesen, M. Locatelli, F. Biella, R. Silipigni, V. Bollati, N. Bresolin, G. Pietro Comi, P. Van Damme, M. Nizzardo, S. Corti, Insights into the identification of a molecular signature for amyotrophic lateral sclerosis exploiting integrated microRNA profiling of iPSC-derived motor neurons and exosomes, Cellular and Molecular Life Sciences 79 (2022) 189. https://doi.org/10.1007/s00018-022-04217-1.

[158] Q. Xu, Y. Zhao, X. Zhou, J. Luan, Y. Cui, J. Han, Comparison of the extraction and determination of serum exosome and miRNA in serum and the detection of miR-27a-3p in serum exosome of ALS patients, Intractable Rare Dis Res 7 (2018) 13–18. https://doi.org/10.5582/irdr.2017.01091.

[159] N. Hayashi, H. Doi, Y. Kurata, H. Kagawa, Y. Atobe, K. Funakoshi, M. Tada, A. Katsumoto, K. Tanaka, M. Kunii, H. Nakamura, K. Takahashi, H. Takeuchi, S. Koyano, Y. Kimura, H. Hirano, F. Tanaka, Proteomic analysis of exosome-enriched fractions derived from cerebrospinal fluid of amyotrophic lateral sclerosis patients, Neurosci Res 160 (2020) 43–49. https://doi.org/10.1016/j.neures.2019.10.010.

[160] Y. Kitamura, M. Kojima, T. Kurosawa, R. Sasaki, S. Ichihara, Y. Hiraku, H. Tomimoto, M. Murata, S. Oikawa, Proteomic Profiling of Exosomal Proteins for Blood-based Biomarkers in Parkinson's Disease, Neuroscience 392 (2018) 121–128. https://doi.org/10.1016/j.neuroscience.2018.09.017.

[161] S. Wang, K. Kojima, J.A. Mobley, A.B. West, Proteomic analysis of urinary extracellular vesicles reveal biomarkers for neurologic disease, EBioMedicine 45 (2019) 351–361. https://doi.org/10.1016/j.ebiom.2019.06.021.

[162] D.H. Ho, S. Yi, H. Seo, I. Son, W. Seol, Increased DJ-1 in Urine Exosome of Korean Males with Parkinson's Disease, Biomed Res Int 2014 (2014) 1–8. https://doi.org/10.1155/2014/704678.

[163] J. Lamontagne-Proulx, I. St-Amour, R. Labib, J. Pilon, H.L. Denis, N. Cloutier, F. Roux-Dalvai, A.T. Vincent, S.L. Mason, C. Williams-Gray, A.-C. Duchez, A. Droit, S. Lacroix, N. Dupré, M. Langlois, S. Chouinard, M. Panisset, R.A. Barker, E. Boilard, F. Cicchetti, Portrait of blood-derived extracellular vesicles in patients with Parkinson's disease, Neurobiol Dis 124 (2019) 163–175. https://doi.org/10.1016/j.nbd.2018.11.002.

[164] Z. Li, D. Chen, R. Pan, Y. Zhong, T. Zhong, Z. Jiao, microRNAs profiling of small extracellular vesicles from midbrain tissue of Parkinson's disease, Front Mol Neurosci 16 (2023). https://doi.org/10.3389/fnmol.2023.1090556.

[165] S. Xie, W. Niu, F. Xu, Y. Wang, S. Hu, C. Niu, Differential expression and significance of miRNAs in plasma extracellular vesicles of patients with Parkinson's disease, International Journal of Neuroscience 132 (2022) 673–688. https://doi.org/10.1080/00207454.2020.1835899.

[166] S.-Y. Chou, L. Chan, C.-C. Chung, J.-Y. Chiu, Y.-C. Hsieh, C.-T. Hong, Altered Insulin Receptor Substrate 1 Phosphorylation in Blood Neuron-Derived Extracellular Vesicles From Patients With Parkinson's Disease, Front Cell Dev Biol 8 (2020). https://doi.org/10.3389/fcell.2020.564641.

[167] M. Hadisurya, L. Li, K. Kuwaranancharoen, X. Wu, Z.-C. Lee, R.N. Alcalay, S. Padmanabhan, W.A. Tao, A. Iliuk, Quantitative proteomics and phosphoproteomics of urinary extracellular vesicles define putative diagnostic biosignatures for Parkinson's disease, Communications Medicine 3 (2023) 64. https://doi.org/10.1038/s43856-023-00294-w.

[168] H.B. Taha, S. Hornung, S. Dutta, L. Fenwick, O. Lahgui, K. Howe, N. Elabed, I. del Rosario, D.Y. Wong, A. Duarte Folle, D. Markovic, J.-A. Palma, U.J. Kang, R.N. Alcalay, M. Sklerov, H. Kaufmann, B.L. Fogel, J.M. Bronstein, B. Ritz, G. Bitan, Toward a biomarker panel measured in CNS-originating extracellular vesicles for improved differential diagnosis of Parkinson's disease and multiple system atrophy, Transl Neurodegener 12 (2023) 14. https://doi.org/10.1186/s40035-023-00346-0.

[169] Z. Wang, Y. Zheng, H. Cai, C. Yang, S. Li, H. Lv, T. Feng, Z. Yu, Aβ1-42-containing platelet-derived extracellular vesicle is associated with cognitive decline in Parkinson's disease, Front Aging Neurosci 15 (2023). https://doi.org/10.3389/fnagi.2023.1170663.

[170] I. Grossi, A. Radeghieri, L. Paolini, V. Porrini, A. Pilotto, A. Padovani, A. Marengoni, A. Barbon, A. Bellucci, M. Pizzi, A. Salvi, G. De Petro, MicroRNA-34a-5p expression in the plasma and in its extracellular vesicle fractions in subjects with Parkinson's disease: An exploratory study, Int J Mol Med 47 (2020) 533–546. https://doi.org/10.3892/ijmm.2020.4806.
